# Neuromuscular function of the quadriceps muscle during isometric maximal, submaximal and submaximal fatiguing voluntary contractions in knee osteoarthrosis patients

**DOI:** 10.1371/journal.pone.0176976

**Published:** 2017-05-15

**Authors:** Anett Mau-Moeller, Robert Jacksteit, Mario Jackszis, Frank Feldhege, Matthias Weippert, Wolfram Mittelmeier, Rainer Bader, Ralf Skripitz, Martin Behrens

**Affiliations:** 1 Department of Orthopaedics, University Medicine Rostock, Rostock, Germany; 2 Institute of Sports Science, University of Rostock, Rostock, Germany; INSEP, FRANCE

## Abstract

**Introduction:**

Knee osteoarthrosis (KOA) is commonly associated with a dysfunction of the quadriceps muscle which contributes to alterations in motor performance. The underlying neuromuscular mechanisms of muscle dysfunction are not fully understood. The main objective of this study was to analyze how KOA affects neuromuscular function of the quadriceps muscle during different contraction intensities.

**Materials and methods:**

The following parameters were assessed in 20 patients and 20 healthy controls: (i) joint position sense, i.e. position control (mean absolute error, MAE) at 30° and 50° of knee flexion, (ii) simple reaction time task performance, (iii) isometric maximal voluntary torque (IMVT) and root mean square of the EMG signal (RMS-EMG), (iv) torque control, i.e. accuracy (MAE), absolute fluctuation (standard deviation, SD), relative fluctuation (coefficient of variation, CV) and periodicity (mean frequency, MNF) of the torque signal at 20%, 40% and 60% IMVT, (v) EMG-torque relationship at 20%, 40% and 60% IMVT and (vi) performance fatigability, i.e. time to task failure (TTF) at 40% IMVT.

**Results:**

Compared to the control group, the KOA group displayed: (i) significantly higher MAE of the angle signal at 30° (99.3%; *P* = 0.027) and 50° (147.9%; *P* < 0.001), (ii) no significant differences in reaction time, (iii) significantly lower IMVT (-41.6%; *P* = 0.001) and tendentially lower RMS-EMG of the rectus femoris (-33.7%; *P* = 0.054), (iv) tendentially higher MAE of the torque signal at 20% IMVT (65.9%; *P* = 0.068), significantly lower SD of the torque signal at all three torque levels and greater MNF at 60% IMVT (44.8%; *P* = 0.018), (v) significantly increased RMS-EMG of the vastus lateralis at 20% (70.8%; *P* = 0.003) and 40% IMVT (33.3%; *P* = 0.034), significantly lower RMS-EMG of the biceps femoris at 20% (-63.6%; *P* = 0.044) and 40% IMVT (-41.3%; *P* = 0.028) and tendentially lower at 60% IMVT (-24.3%; *P* = 0.075) and (vi) significantly shorter TTF (-51.1%; *P* = 0.049).

**Conclusion:**

KOA is not only associated with a deterioration of IMVT and neuromuscular activation, but also with an impaired position and torque control at submaximal torque levels, an altered EMG-torque relationship and a higher performance fatigability of the quadriceps muscle. It is recommended that the rehabilitation includes strengthening and fatiguing exercises at maximal and submaximal force levels.

## Introduction

Knee osteoarthrosis (KOA) is commonly associated with a dysfunction of the quadriceps muscle which mainly contributes to alterations in motor performance [1, 2]. The quadriceps muscle is functionally important for knee joint stabilization and a persistent weakness reduces the protective force generated at the knee joint. Sensorimotor dysfunction of the quadriceps muscle is even regarded as being associated with progression of KOA [1]. It has been shown that the restoration and improvement of muscle function lead to improved symptoms and joint structure [2], and there is a growing body of evidence showing that exercise can improve muscle strength [3], physical function [4] and walking ability [5] in KOA patients. Furthermore, studies have shown that preoperative quadriceps strength is a predictor for postoperative muscle strength and functional outcome following total knee arthroplasty [6]. Consequently, current clinical guidelines recommend exercise for the treatment of KOA [7]. However, a successful creation and implementation of specific movement therapy measures implies the understanding of the neuromuscular deficit [8]. There is a gap in the knowledge regarding the neuromuscular mechanisms of quadriceps muscle dysfunction indicating a need for further research. Therefore, the purpose of this study was to investigate how KOA affects neuromuscular control of the quadriceps muscle during different contraction intensities in end-stage KOA patients.

### Joint position sense—Position control

Proprioception is regarded as being important for knee function and prevention of joint damage. It is assumed to be required for coordination and precision of complex movements, stabilization of the knee joint and protection against injurious movements. Impaired proprioceptive accuracy for joint position sense in KOA patients has been found in various studies (for a review see Knoop 2011) [9]. However, Knoop et al. concluded that causes of KOA-related impairments in joint position sense have not been identified.

Furthermore, measurement protocols differ (e.g. sitting, standing or lying position, active or passive (re)positioning, target angle) and most KOA-studies have analyzed joint position sense in sitting position with an hip angle of 90° [9]. In this study, we measured joint position sense in a lying position with a hip angle of 30°. A greater hip angle in lying compared to sitting position may have effects on muscle activation due to changes in rectus femoris muscle length, moment arm length and modulations in neuromuscular function like altered discharge from muscle afferents [10–12].

### Reaction time task performance

Quadriceps muscle dysfunction in KOA patients is associated with a higher risk to sustain a fall [13], which might be partly due to an impaired ability to quickly activate skeletal muscles [14]. There is, however, little research on reaction time task performance in response to a simple visual stimulus in KOA patients. This is the first study on reaction time, premotor time and motor time of the quadriceps muscle in response to a simple visual stimulus in patients with KOA.

### Isometric maximal voluntary contraction–isometric maximal voluntary force/torque and neuromuscular activation

There is comprehensive evidence that KOA is associated with a quadriceps muscle strength deficit during isometric maximal voluntary contraction (IMVC) [15–19] which is partly due to muscle atrophy and partly due to arthrogenic muscle inhibition (AMI) [20]. Quadriceps muscle strength is, however, functionally relevant for motor performance during activities of daily living [21, 22]. To date only a few studies have investigated neuromuscular activation of the quadriceps muscle during IMVC [17, 23–25]. Studies have shown lower values for rectus femoris and vastus medialis muscle activity and no difference in the activation of vastus lateralis compared with healthy controls [24, 25]. However, muscle activity of the quadriceps muscle was also found to be higher in two different knee flexion angles (30° and 60°) during IMVC in KOA patients [23]. Further findings obtained by Stevens-Lapsley et al. demonstrated a higher coactivation of the hamstring muscles [17]. Nevertheless, general conclusions on neuromuscular activation during IMVC in KOA patients cannot be drawn due to the limited number of studies and the contradictory findings. In the present study, rectus femoris, vastus lateralis and biceps femoris muscle activity was analyzed in order to provide further insight into the neuromuscular control during IMVC.

### Isometric submaximal voluntary contraction–force/torque control and EMG-force/torque relationship

Moreover, many other activities of daily living such as standing and walking are predominately performed at submaximal force intensities. To the best of our knowledge, only two studies have investigated neuromuscular activation and motor unit characteristics of the quadriceps muscle at submaximal force levels in KOA patients [18, 26]. Data from these two studies indicate that muscle activity, motor unit recruitment and rate coding strategies are altered. It could be further speculated that these differences in motor unit properties would result in a reduced ability to control force at submaximal levels. The quality of force production can be assessed by analyzing the accuracy, fluctuations and periodicity of the force/torque signal [27–33]. Force/torque at a given submaximal voluntary contraction is not constant and fluctuates around a mean. Fluctuation in motor output is functionally relevant for the accuracy of movement and has been identified as a predictor of performance during functional tasks (chair rise and stair climbing) [34]. It can be assumed that force production executed with less control could accelerate joint damage in subjects with KOA, particularly in combination with an impaired sense of position [9].

To the best of our knowledge, only two studies have investigated force control in KOA and they showed modulations depending on the contraction type [28, 35]. Hortobagyi et al. investigated force fluctuations at two fixed force levels (50 N and 100 N) which, however, did not represent the same relative target forces in % of IMVC strength of both groups [28]. Smith et al. measured force control at 50% of IMVC strength during concentric and eccentric voluntary contractions [35]. We present the first study on torque control at 20%, 40% and 60% of IMVC strength.

Furthermore, neuromuscular activation during these different submaximal torque levels was analyzed. This aspect has been explicitly addressed in only two studies and they revealed contrary findings [18, 26]. Thus, the present data may provide further information on neuromuscular activation during isometric submaximal voluntary contractions in KOA patients.

### Isometric submaximal fatiguing voluntary contraction—Performance fatigability

Muscle weakness is generally associated with a reduced fatigue resistance [36]. To the extent of our knowledge, only two studies have investigated performance fatigability during and following IMVCs in KOA patients [37, 38]. Although these two studies have indicated that fatigue resistance during and following IMVCs is reduced in KOA patients, no attention has been given to performance fatigability during submaximal voluntary contraction which, however, is relevant for performing everyday activities. In the present study, for the first time, fatigue resistance was analyzed during isometric submaximal fatiguing voluntary contraction at 40% of IMVT.

In conclusion, in order to provide a comprehensive overview of modulations in neuromuscular function associated with KOA, the present study analyzed (i) joint position sense, i.e. position control at 30° and 50° of knee flexion, (ii) simple reaction time task performance, (iii) IMVT and average EMG of rectus femoris, vastus lateralis and biceps femoris, (iv) torque control, i.e. accuracy, absolute fluctuation, relative fluctuation and periodicity of the torque signal at 20%, 40% and 60% IMVT, (v) EMG-torque relationship at 20%, 40% and 60% IMVT and (vi) performance fatigability, i.e. time to task failure at 40% IMVT.

## Materials and methods

### Ethics statement

The cross-sectional study was carried out in the Department of Orthopaedics of the University Medicine Rostock, Germany from January 2014 to December 2014. The study was approved by the Ethical Review Committee of the University of Rostock (A 2013–0150).

### Participants

Twenty healthy subjects with no history of neurological and musculoskeletal disorders/injuries and 20 subjects with KOA scheduled for primary total knee arthroplasty surgery volunteered for this study. Patients were identified as suitable for inclusion if they were aged between 50 and 80 and had a body mass index of less than 40 kg/m^2^. Patients with a total knee endoprosthesis on the contralateral side or a total hip endoprosthesis were enrolled in this study when the operation had taken place at least one year before. The following exclusion criteria were defined for the patient group: musculoskeletal disorders besides KOA, neurological disorders, metabolic bone disease and pain or functional restrictions that would prevent patients from taking part in physical examinations. Prior to taking part in the study all participants signed a declaration of consent. The demographic and clinical characteristics of the participants are provided (Table 1).

**Table 1:** Demographic and clinical subject characteristics.

	Patient group (n = 20)	Control group (n = 20)	*P*
Age (yrs)	66.7 (8.8)	62.1 (6.2)	0.066^†^
Sex (men)	7.0 (35%)	5.0 (25%)	0.490
Weight (kg)	91.3 (17.4)	71.5 (14.3)	< 0.001**
Height (m)	1.68 (0.10)	1.67 (0.10)	0.901
BMI (kg/m^2^)	32.5 (5.8)	25.4 (3.5)	< 0.001**
Physical activity (h/week)	0.25 (0.64)	0.95 (1.20)	0.029*
Knee pain^‡^ during…			
• rest	5.3 (2.4)	0.0	
• simple reaction time task	2.0 (2.1)	0.0	
• isometric maximum voluntary contractions	4.8 (2.7)	0.0	
• isometric submaximal voluntary contractions	3.5 (2.4)	0.0	
• isometric submaximal fatiguing voluntary contraction	3.7 (2.2)	0.0	

Values are presented as means (standard deviation) or numbers (%)

Abbreviations: BMI, body mass index.

* denotes a significant difference between groups (** *P* ≤ 0.010).

^†^ denotes a statistical tendency towards a difference between groups (*P* ≤ 0.100).

^‡^ visual analogue scale (0–10)

### Experimental design and procedures

The study design encompassed one measurement point in time (two-hour session). Patients were examined one day before total knee arthroplasty. Neuromuscular function of the quadriceps muscle was measured unilaterally on the right leg of the control subjects and the affected side of the patients (right leg: n = 11 (55%)). Leg dominance was not controlled for in data analyses; this might be a confounding factor. The data were collected by the same investigator.

#### Pain

Pain was investigated using a 10 cm visual analogue scale [39]. Participants were asked to mark their perceived knee pain on a continuous horizontal scale whereby the very left end indicated no pain (score 0) and the very right end (score 10) indicated unbearable pain. Measurements included (i) the subjective knee pain score at rest in supine position and (ii) pain present during measurements, i.e. while performing the simple reaction time task, IMVC, isometric submaximal voluntary contractions and isometric submaximal fatiguing voluntary contraction.

#### Joint position sense

**Fig 1:**
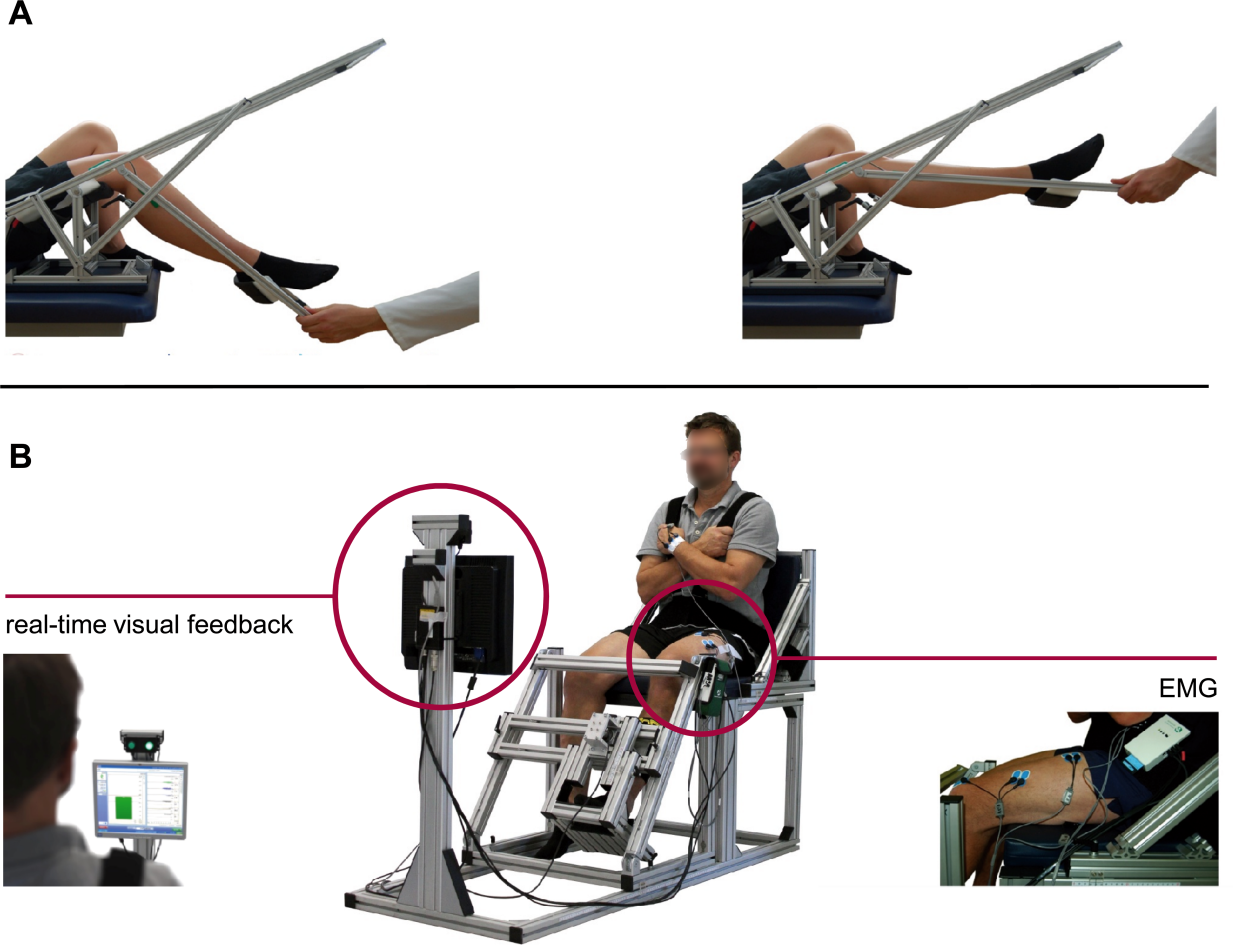
Custom-made systems for measuring (A) joint position sense and (B) knee extension torque.

Knee joint position sense was tested by measuring the subjects’ ability to actively reproduce a previously presented knee flexion angle. The joint repositioning test was carried out by utilizing a custom-made splint modified according to Barrett et al. and Jerosch et al. [40, 41]. The splint consists of aluminium profiles (item, Industrietechnik GmbH, Solingen, Germany) and well-cushioned surfaces for supporting the leg (Fig 1A). Subjects lay in a supine position with one leg in the splint and the hip joint at 30°. Lin et al. found a between-session intrarater intraclass correlation coefficient (ICC) of 0.84 for the measurement of joint position sense in supine position indicating moderate reliability [42]. The starting position was defined at 70° knee flexion (0° = full extension). The subjects’ extension/flexion axes of the knee were aligned with the axis of the splint. Measurements were carried out in a quiet room without any auditive interference. Visual cues were eliminated by wearing a completely darkened safety goggle (MSA Auer GmbH, Berlin, Germany).

Knee angle was measured using a twin-axis electrogoniometer (SG 150, Biometrics Ltd, Newport, United Kingdom). One endblock of the goniometer was placed on the aluminium profile parallel to the longitudinal axis of the femur in line with the greater trochanter and the lateral femoral condyle. The other end was applied to the skin with double-sided tape parallel to the tibia in line with the fibular head and the lateral malleolus. Signals were amplified, band-pass filtered (10–450 Hz) and digitized with the Telemyo 2400T G2 eight-channel EMG telemetry system (Noraxon Inc., Scottsdale, AZ, USA). Data were sampled at 3 kHz.

The shank was moved passively by an observer from the starting position at 70° knee flexion to two different predetermined knee flexion angles (30° and 50°). The respective target angle was kept for 15 s and the subjects were instructed to concentrate on the position of the leg in space and remember the specific position. Afterwards, the shank was returned to the starting position and the subjects had to actively reproduce the previously presented knee angle and hold the position for 5 s. The subjects performed one familiarization trial and five test trials for each knee flexion angle in a random order with a rest interval of 15 s between the trials. From each trial, the ascending and descending part of the angle signal as well as the first 15% and last 15% of the plateau phase were removed. Position accuracy was assessed as mean absolute error (MAE) between the target and reproduced knee flexion angle.

#### Torque and EMG measurements

**Fig 2:**
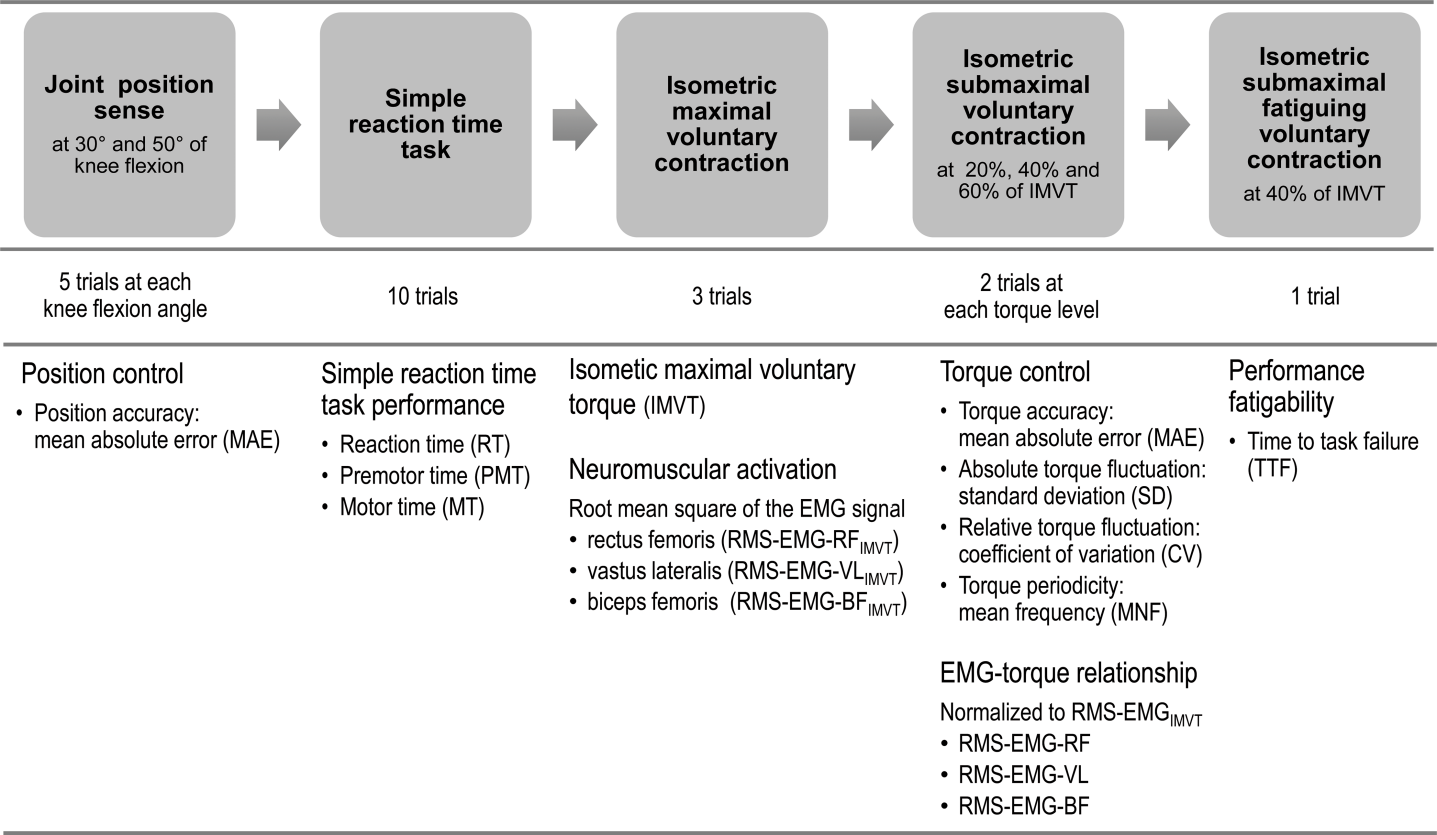
Schematic illustration of the test protocol.

Torque and EMG recordings were performed during (i) the simple reaction time task, (ii) IMVC, (iii) isometric submaximal voluntary contraction, and (iv) isometric submaximal fatiguing voluntary contraction. Measurements were carried out in the sequence as described above. Fig 2 shows the order and parameters of torque and EMG measurements.

The knee extension torque measurements were carried out using a custom-made measurement system. It is designed according to the principle of a knee-extension machine and allows the seating position to be individually adjusted (Fig 1B). The measurements were performed unilaterally with the hip joint (90°), ankle joint (90°) and knee joint (60–70°; 0° = full extension) all at constant angles. Velcro straps across the waist and shoulder prevented excessive movements of the trunk. The shank was fixed to a panel 2–3 cm above the lateral malleolus. The patients were instructed to fold their arms across their chests and to extend the leg isometrically against the panel. The torque signal was captured with a KM40 force sensor (ME-Messsysteme GmbH, Hennigsdorf, Germany), preamplified (GSV3, ME-Messsysteme GmbH, Hennigsdorf, Germany) and recorded at a sampling rate of 3 kHz with the Telemyo 2400T G2 EMG telemetry system. The signals were filtered using MATLAB (version R2012b; The MathWorks, Inc., Natick, MA, USA): third-order Butterworth IIR low-pass filter (25 Hz). The torque was calculated by multiplying the force to the length of the lever arm.

Surface EMG was recorded using bipolar EMG Ambu^®^ Blue Sensor N electrodes (2 cm diameter). The electrodes were firmly attached to the shaved, abraded and cleaned skin over the rectus femoris, vastus lateralis and biceps femoris. Electrical resistance between electrodes was measured with a digital multimeter (MY-68, McVoice, Braunschweig, Germany) and was kept below 5 kΩ. The electrodes were applied with a centre-to-centre distance of 2 cm over the middle of the muscle bellies. The recording electrodes were in line with the presumed direction of the underlying muscle fibres and the reference electrode was attached to the patella of the ipsilateral leg. Signals were amplified and digitized with the Telemyo 2400T G2 EMG telemetry system (sampling rate 3 kHz). The signals were filtered using MATLAB: (1) third-order Butterworth IIR band-pass filter (10–450 Hz); (2) IIR notch filter (50 Hz).

#### Simple reaction time task

The subjects sat on the measurement system and faced a horizontal black box (length 12 cm x height 6 cm) with two green light-emitting diodes (LEDs) positioned 110 cm in front of them at eye level. The LEDs had a diameter of 2.7 cm and were fixed 4.5 cm apart. For the measurement of simple reaction time, patients were instructed to extend the leg isometrically as quickly and accurately as possible when both green LEDs lit up. Thus, patients performed only very short contractions (duration: ~1 s) at low force levels. The duration of the light stimulus was 1 s and the interstimulus interval varied randomly between 5 and 15 s. No familiarization was carried out. The patients performed ten test trials. Reaction time, premotor time and motor time were analyzed. Reaction time was measured as the interval between stimulus onset and the onset of motor response (torque onset). Premotor time was defined as the time interval between stimulus onset and EMG onset and motor time was measured as the time interval between EMG onset and torque onset [43]. Torque and EMG onsets were identified manually, as described previously [44]. The mean value of the ten test trials and the fastest trial were used for data analysis. Trials with anticipated and delayed responses, i.e. premotor time outside the range of 100–500 ms, were excluded from the analysis.

#### Isometric maximal voluntary contraction

The IMVT was measured by asking the subjects to exert isometric maximal voluntary knee extensions against a panel for 3 s. The patients were instructed to act as forcefully as possible and were given strong verbal encouragement throughout the execution of the movement. An observer checked that the movement was performed without any visible countermovement or pre-tension. The patients performed three to five familiarization trials. When the coefficient of variation (CV) of three subsequent trials was below 5% [45], three test trials were performed. The rest time between the trials was 1 min. Subjects received feedback on their strength performance after each trial. The mean value of the three test trials was used to determine IMVT and muscle activity. The EMG signals were rectified, filtered and averaged (root mean square, RMS) over a 200 ms period at IMVT: 100 ms preceding and 100 ms following the IMVT (rectus femoris: RMS-EMG-RF_IMVT_; vastus lateralis: RMS-EMG-VL_IMVT_; biceps femoris: RMS-EMG-BF_IMVT_) [46].

#### Isometric submaximal voluntary contraction

Subjects performed isometric voluntary contractions for a duration of 12 s at three different submaximal torque levels: 20%, 40%, and 60% of IMVT. The target and actual torques were displayed as real-time visual feedback on a 19-inch monitor which was located 110 cm in front of the subject. The actual torque was provided as a vertical bar graph and the target torque as a horizontal line. Two trials were performed at each torque level in random order with a minimum of 1 min rest interval between the contractions.

From each trial, the ascending and descending parts of the torque signal were removed and a period of 8 s in the middle of the isometric contraction, when the actual torque fluctuated around the target torque, was analyzed [29]. Torque accuracy was expressed as MAE of the actual torque relative to the target torque in percent of the target torque level [27, 28]. The standard deviation (SD) and CV of the torque signal were calculated and represent the absolute and relative torque fluctuation, respectively [29, 30]. The mean frequency (MNF) of the torque signal was analyzed in order to estimate torque periodicity [31–33]. Furthermore, RMS-EMG over a 1 s period in the middle of the 8 s period was normalized to their respective RMS-EMG_IMVT_ [47]. The mean value of the two test trials at each torque level was used for data analysis.

#### Isometric submaximal fatiguing voluntary contraction

Subjects performed a sustained submaximal fatiguing voluntary contraction until task failure. Real-time visual feedback of the target and actual torque was provided as a horizontal line and a vertical bar graph, respectively, on a 19-inch monitor at a distance of 110 cm in front of the subjects. During the task, subjects were asked to maintain a consistent torque at 40% of IMVT as long and as accurately as possible. The criterion for task failure was the inability to keep the torque within 10% of the target torque for > 3 s, despite strong verbal encouragement. The time to task failure was analyzed.

### Statistical analysis

Data were checked for normal distribution using the Kolmogorov-Smirnov test. Differences between the groups were tested for significance with a chi-squared test (parameter: sex), unpaired Student’s *t* tests (parameter: age, height, weight, BMI and physical activity) or analyzes of covariance (ANCOVA) adjusted for age, gender, weight and height (all parameters of neuromuscular function). The level of significance was set at *P* ≤ 0.050. All data were analyzed using the SPSS statistical package 20.0 (SPSS Inc., Chicago, IL, USA). Data are provided in the Supporting Information file (S1 Dataset). The effect size was calculated with G*Power (version 3.1.4.) and interpreted using Cohen’s classification: *f* = 0.10 small effect, *f* = 0.25 moderate effect, *f* = 0.40 large effect [48]. Data are presented as adjusted mean values (adjusted SD) and adjusted mean difference (95% confidence interval [95% CI]).

Furthermore, the intra-session reliability of IMVT was calculated using the two trials with the highest peak torque. The typical error of measurement (TE) and coefficient of variation (CV) were calculated to provide measures of absolute reliability, representing the degree of variability in repeated measurements for a given individual (within-subject variation) [49]. A CV of ≤ 10% was defined as high reliability [50]. Relative reliability was estimated using the ICC with 95% confidence level that represents the variation of the rank order of all the subjects in a re-test [49]. ICC values were classified as follows: value ≥ 0.90 were considered high, values between 0.80 and 0.90 as moderate and values ≤ 0.80 as low [51]. Between-trial differences were assessed with paired Student’s *t* tests. Data were analyzed using an Excel spreadsheet developed by Hopkins [52] and the SPSS statistical package 20.0. Absolute and relative intra-session reliability of IMVT was high, with ICC values of 0.99 and CV values below 5.2%. A summary of the reliability data is given in Table 2.

**Table 2:** Intra-session reliability of isometric maximal voluntary torque (N∙m) of the knee extensors in knee osteoarthrosis patients (n = 20) and healthy controls (n = 20).

	Trial 1 Mean (SD)	Trial 2 Mean (SD)	Mean difference (95% CI)	SD_Diff_	TE (95%CI)	CV (95% CI)	ICC (95% CI)
**Patient group**	110.3 (58.5)	108.0 (58.5)	-2.29 (-5.42 to 0.83)	6.68	4.72 (3.59 to 6.90)	5.20 (3.97 to 7.76)	0.99 (0.99 to 1.0)
**Control group**	162.6 (50.0)	162.1 (48.0)	-0.52 (-4.55 to 3.51)	8.60	6.08 (4.63 to 8.89)	3.76 (2.84 to 5.53)	0.99 (0.97 to 0.99)

Abbreviations: SD, standard deviation; SD_Diff_, SD of the difference between trial 1 and 2; TE, typical error; CV, coefficient of variation; ICC, intra-class correlation coefficient.

## Results

### Joint position sense

The MAE during the knee joint repositioning test was significantly higher at 30° (99.3%) and 50° (147.9%) of knee flexion in patients than in controls (Table 3). Furthermore, an angle-specific difference in MAE was found; i.e. patients revealed a significantly higher MAE in 50° compared to 30° of knee flexion (mean difference: 3.68°; 95% CI: 0.57 to 6.78; *P* = 0.023) whereas no difference was found in the control group (mean difference: 0.81°; 95% CI: -2.31 to 3.94; *P* = 0.592).

**Table 3:** Data of knee joint position sense and simple reaction time task in knee osteoarthrosis patients (n = 20) and healthy controls (n = 20).

	Patient group Mean (SD)^‡^	Control group Mean (SD) ^‡^	Mean difference (95% CI)	*F*	*P*	*η*_*p*_^*2*^	*f*	*Power*
**Joint position sense (°)**								
• MAE 50° knee flexion	17.6 (7.1)	7.1 (7.0)	10.5 (5.0 to 16.0)	15.27	< 0.001**	0.323	0.691	0.989
• MAE 30° knee flexion	13.4 (7.7)	6.7 (7.5)	6.7 (0.8 to 12.6)	5.36	0.027*	0.143	0.408	0.709
**Simple reaction time task (s)**								
Mean of 10 trials								
• Reaction time	0.281 (0.072)	0.284 (0.074)	-0.002 (-0.057 to 0.053)	0.01	0.929	< 0.001	< 0.032	< 0.054
• Premotor time rectus femoris	0.211 (0.058)	0.208 (0.058)	0.003 (-0.041 to 0.047)	0.02	0.884	0.001	0.032	0.054
• Premotor time vastus lateralis	0.220 (0.062)	0.222 (0.062)	-0.002 (-0.047 to 0.043)	0.01	0.933	< 0.001	< 0.032	< 0.054
• Motor time rectus femoris	0.076 (0.045)	0.081 (0.045)	-0.004 (-0.037 to 0.029)	0.07	0.798	0.002	0.045	0.059
• Motor time vastus lateralis	0.061 (0.040)	0.068 (0.040)	-0.007 (-0.035 to 0.022)	0.24	0.622	0.007	0.084	0.081
Fastest trial								
• Reaction time	0.217 (0.049)	0.223 (0.049)	-0.006 (-0.043 to 0.031)	0.11	0.743	0.003	0.055	0.063
• Premotor time rectus femoris	0.158 (0.054)	0.159 (0.054)	-0.022 (-0.040 to 0.037)	0.01	0.935	< 0.001	< 0.032	< 0.054
• Premotor time vastus lateralis	0.171 (0.089)	0.150 (0.089)	0.021 (-0.019 to 0.062)	1.13	0.295	0.032	0.181	0.201
• Motor time rectus femoris	0.065 (0.358)	0.061 (0.358)	0.004 (-0.023 to 0.031)	0.09	0.763	0.003	0.055	0.063
• Motor time vastus lateralis	0.051 (0.058)	0.071 (0.058)	-0.021 (-0.062 to 0.020)	1.05	0.313	0.030	0.176	0.191

Abbreviations: MAE, mean absolute error.

* denotes a significant difference between groups (* *P* ≤ 0.050; ** *P* ≤ 0.010).

^‡^ Values are presented as estimated marginal means (standard deviation): ANCOVA adjusted for sex, weight, height and age.

### Simple reaction time task

No significant differences in reaction time, premotor time and motor time during the simple reaction time task were found (Table 3).

### Isometric maximal voluntary contraction

KOA patients produced significantly lower IMVT (-41.6%) accompanied by a tendentially reduced RMS-EMG of the rectus femoris (-33.7%) than control subjects. No significant group differences were found for the RMS-EMG of the vastus lateralis and biceps femoris (Table 4).

**Table 4:** Data of isometric maximal, submaximal and submaximal fatiguing voluntary contractions in knee osteoarthrosis patients (n = 20) and healthy controls (n = 20).

	Patient group Mean (SD) ^‡^	Control group Mean (SD) ^‡^	Mean difference (95% CI)	*F*	*P*	*η*_*p*_^*2*^	*f*	*Power*
**Isometric maximal voluntary contraction**								
• IMVT (N∙m)	98.5 (51.8)	168.6 (51.8)	-70.1 (-108.2 to -32.0)	13.99	0.001**	0.292	0.642	0.976
• RMS-EMG-RF_IMVT_ (μV)	77.6 (54.0)	117.0 (54.0)	-39.4 (-79.5 to 0.66)	4.00	0.054^†^	0.105	0.343	0.558
• RMS-EMG-VL_IMVT_ (μV)	115.9 (83.3)	144.3 (83.3)	-28.4 (-89.6 to 32,8)	0.89	0.352	0.026	0.163	0.171
• RMS-EMG-BF_IMVT_ (μV)	26.8 (15.4)	20.5 (15.4)	6.3 (-5.0 to 17.6)	1.28	0.266	0.036	0.193	0.221
**Isometric submaximal voluntary contraction (EMG-torque relationship)**								
RMS-EMG-RF (%RMS-EMG-RF_IMVT_)								
• 20% IMVT	24.9 (14.1)	19.2 (14.1)	5.7 (-4.7 to 16.1)	1.26	0.270	0.036	0.193	0.221
• 40% IMVT	39.6 (16.9)	36.0 (16.9)	3.7 (-8.8 to 16.1)	0.36	0.553	0.010	0.101	0.095
• 60% IMVT	59.8 (23.4)	57.8 (23.4)	2.0 (-15.2 to 19.2)	0.06	0.815	0.002	0.045	0.059
RMS-EMG-VL (%RMS-EMG-VL_IMVT_)								
• 20% IMVT	27.5 (10.0)	16.1 (10.0)	11.4 (4.1 to 18.8)	10.01	0.003**	0.227	0.542	0.915
• 40% IMVT	43.6 (13.6)	32.7 (13.6)	10.9 (0.9 to 20.9)	4.89	0.034*	0.126	0.380	0.646
• 60% IMVT	61.4 (17.6)	52.5 (17.6)	8.9 (-4.1 to 21.8)	1.94	0.172	0.054	0.239	0.312
RMS-EMG-BF (%RMS-EMG-BF_IMVT_)								
• 20% IMVT	19.3 (44.7)	53.0 (44.7)	-33.8 (-66.6 to -0.9)	4.36	0.044*	0.114	0.359	0.597
• 40% IMVT	33.3 (28.3)	56.7 (28.3)	-23.4 (-44.2 to -2.7)	5.27	0.028*	0.134	0.393	0.676
• 60% IMVT	50.9 (24.6)	67.2 (24.6)	-16.3 (-34.4 to 1.8)	3.37	0.075^†^	0.090	0.314	0.489
**Isometric submaximal fatiguing voluntary contraction**								
Time to task failure (s)	178 (251)	364 (251)	-185 (-370 to -1)	4.17	0.049*	0.109	0.350	0.575

Abbreviations: IMVT, isometric maximal voluntary torque; RMS-EMG, root means square of the EMG signal; RF, rectus femoris; VL, vastus lateralis; BF, biceps femoris; %RMS-EMG, RMS-EMG normalized to its respective RMS-EMG during IMVT.

* denotes a significant difference between groups (*P ≤ 0.050; ** *P* ≤ 0.010).

^†^ denotes a statistical tendency towards a difference between groups (*P* ≤ 0.100).

^‡^ Values are presented as estimated marginal means (standard deviation): ANCOVA adjusted for sex, weight, height and age.

### Isometric submaximal voluntary contraction

**Fig 3:**
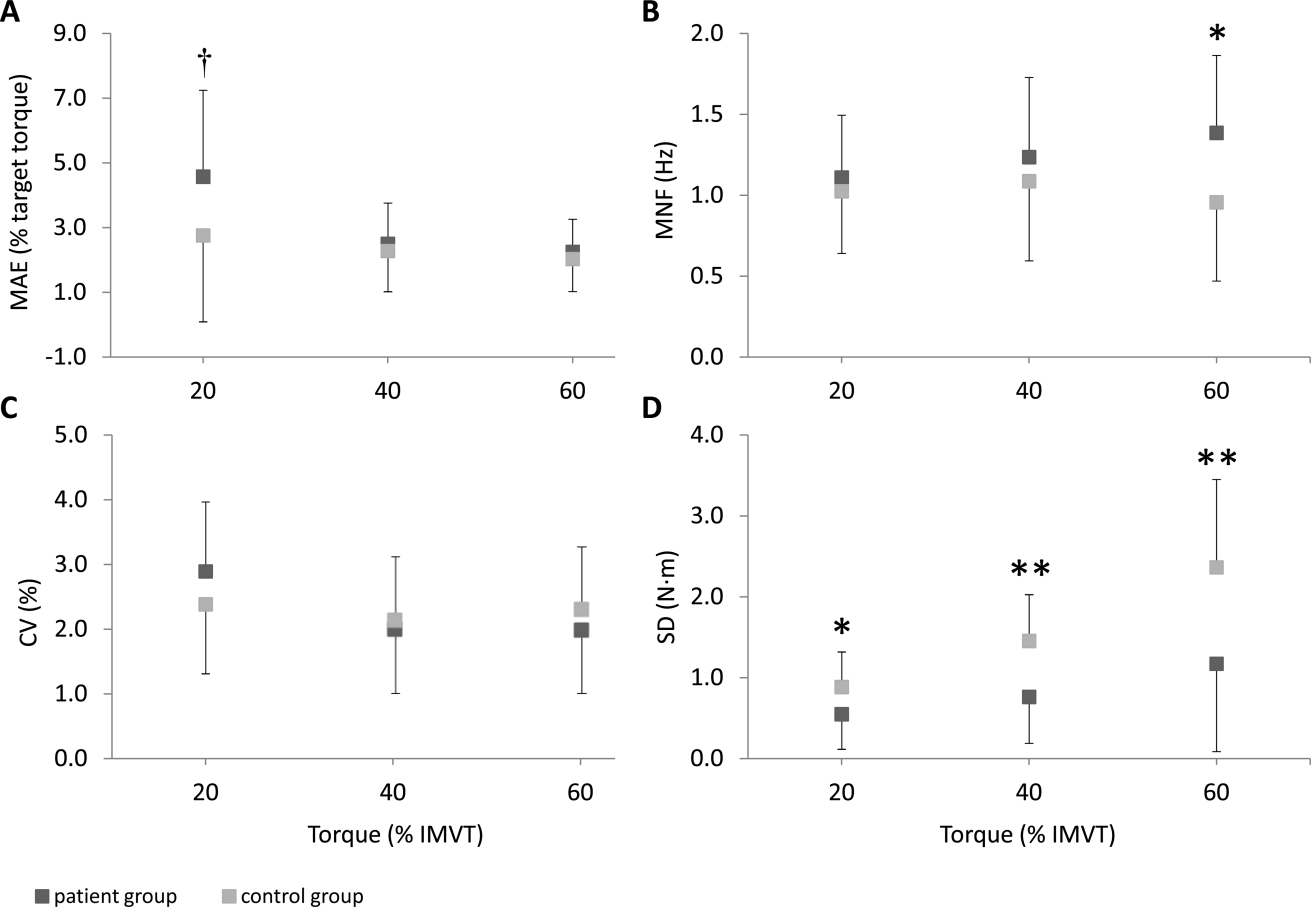
Torque control during 20%, 40% and 60% of isometric maximal voluntary torque (IMVT). **(A) MAE, mean absolute error (torque accuracy), (B) MNF, mean frequency (torque periodicity), (C) CV, coefficient of variation (relative torque fluctuation), and (D) SD, standard deviation (absolute torque fluctuation).** ANCOVA adjusted for sex, weight, height and age. * denotes a significant difference between groups (*P ≤ 0.050; ** *P* ≤ 0.010). † denotes a statistical tendency towards a difference between groups (*P* ≤ 0.080).

The MAE of the torque signal (torque accuracy) was tendentially higher in the patient group by 65.9% at 20% of IMVT. No significant between-group differences were found at 40% and 60% of IMVT. The SD of the torque signal (absolute torque fluctuation) was significantly reduced at all three torque levels in KOA patients, i.e. -37.9% at 20% of IMVT, -47.6% at 40% of IMVT and -50.4% at 60% of IMVT. There were no significant differences in CV (relative torque fluctuation) between patients and controls. Compared to the control subjects, KOA patients had 44.8% greater MNF (torque periodicity) at 60% of IMVT whereas no significant differences between groups were found at 20% and 40% of IMVT. The results of torque accuracy, absolute and relative torque fluctuations and torque periodicity are displayed in Table 5 and Fig 3.

**Table 5:** Data of torque control during isometric submaximal voluntary contractions in knee osteoarthrosis patients (n = 20) and healthy controls (n = 20).

	Mean difference (95% CI)^‡^	*F*	*P*	*η*_*p*_^*2*^	*f*	*Power*
**Torque accuracy (% target torque level)**						
• MAE 20% IMVT	1.98 (-0.14 to 3.78)	3.55	0.068^†^	0.094	0.322	0.508
• MAE 40% IMVT	0.23 (-0.70 to 1.15)	0.25	0.623	0.007	0.084	0.081
• MAE 60% IMVT	0.22 (-0.52 to 0.96)	0.37	0.547	0.011	0.105	0.099
**Absolute torque fluctuation (N∙m)**						
• SD 20% IMVT	-0.33 (-0.65 to -0.02)	4.57	0.040*	0.119	0.368	0.617
• SD 40% IMVT	-0.69 (-1.11 to -0.27)	11.16	0.002**	0.247	0.573	0.940
• SD 60% IMVT	-1.19 (-1.99 to -0.39)	9.17	0.005**	0.212	0.519	0.890
**Relative torque fluctuation (%)**						
• CV 20% IMVT	0.51 (-0.28 to 1.30)	1.72	0.198	0.048	0.225	0.281
• CV 40% IMVT	-0.14 (-0.85 to 0.56)	0.17	0.682	0.005	0.071	0.072
• CV 60% IMVT	-0.31 (-0.99 to 0.38)	0.82	0.372	0.024	0.157	0.161
**Torque periodicity (Hz)**						
• MNF 20% IMVT	0.08 (-0.20 to 0.37)	0.37	0.550	0.011	0.105	0.099
• MNF 40% IMVT	0.15 (-0.21 to 0.51)	0.70	0.407	0.020	0.143	0.142
• MNF 60% IMVT	0.43 (0.08 to 0.78)	6.19	0.018*	0.154	0.154	0.427

Abbreviations: MAE, mean absolute error; IMVT, isometric maximal voluntary torque; SD, standard deviation; CV, coefficient of variation; MNF, mean frequency.

* denotes a significant difference between groups (*P ≤ 0.050; ** *P* ≤ 0.010).

^†^ denotes a statistical tendency towards a difference between groups (*P* ≤ 0.100).

^‡^ ANCOVA adjusted for sex, weight, height and age.

**Fig 4:**
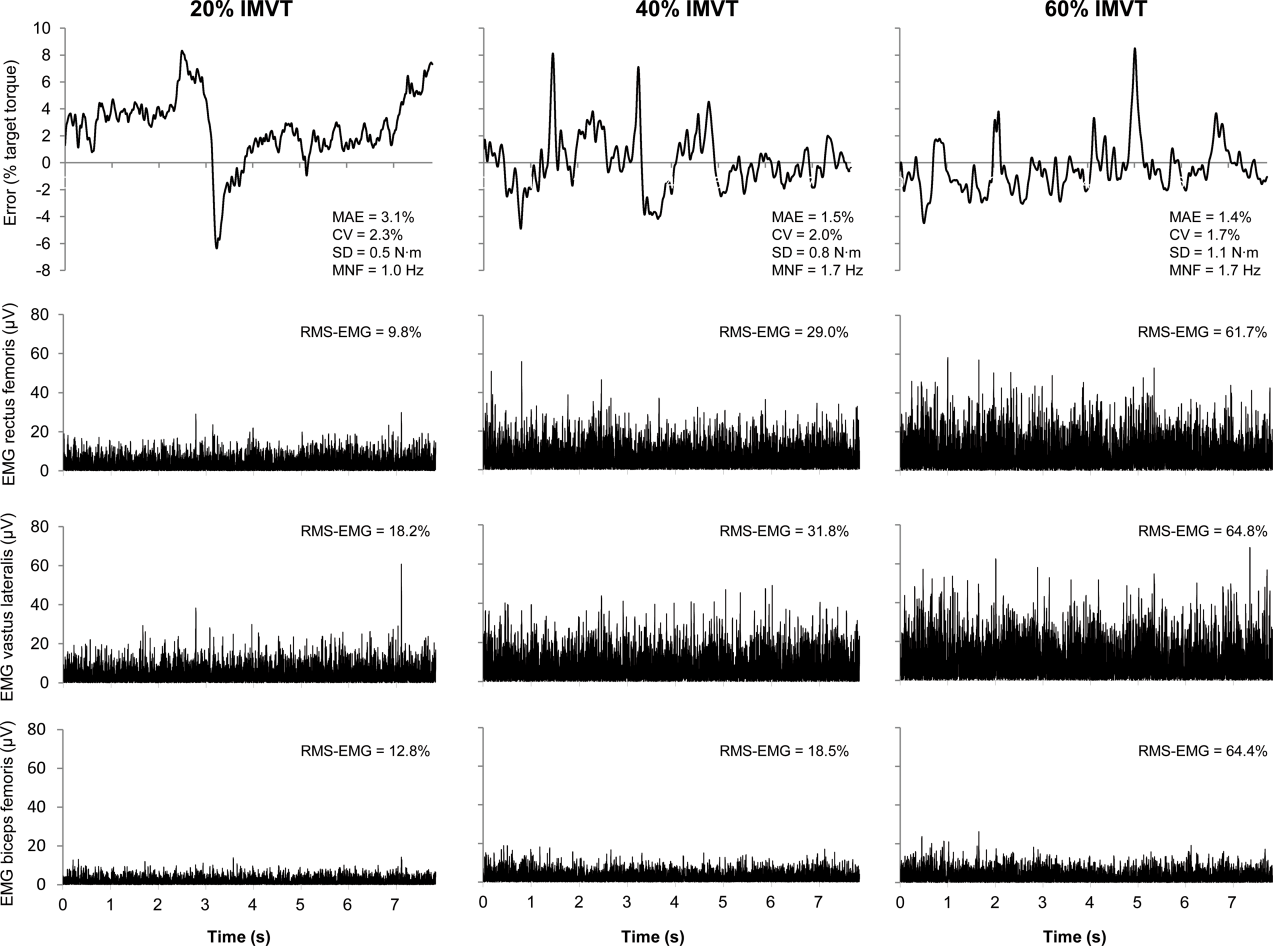
Example data sets of the knee extension torque error (% target torque) and rectified EMG signals from a patient recorded during 20%, 40% and 60% of isometric maximal voluntary torque (IMVT). MAE, mean absolute error; CV, coefficient of variation; SD, standard deviation; MNF, mean frequency; RMS-EMG, root mean square of the EMG signal normalized to its respective RMS-EMG during IMVT.

Between-group differences in muscle activity could be documented for the vastus lateralis at 20% and 40% of IMVT with higher RMS-EMG values (70.8% and 33.3%, respectively) in the patient group. However, biceps femoris RMS-EMG values were significantly lower in the patient group at 20% and 40% of IMVT (-63.6% and -41.3%, respectively) and tendentially lower at 60% of IMVT (-24.3%) (Table 4). Fig 4 shows example data sets from a patient during 20%, 40% and 60% of isometric maximal voluntary torque to illustrate the difference in torque control between torque levels.

### Isometric submaximal fatiguing voluntary contraction

KOA patients produced significantly shorter time to task failure (-51.1%) during sustained submaximal fatiguing voluntary contraction than control subjects (Table 4).

## Discussion

This study provides a contribution to the ongoing discussion about the neuromuscular mechanisms of quadriceps muscle dysfunction in patients with KOA. It has been clearly shown that IMVT and corresponding neuromuscular activation were reduced in end-stage KOA patients compared with healthy control subjects. Furthermore, KOA patients revealed an impaired position and torque control at submaximal torque levels, a modified EMG-torque relationship and a diminished fatigue resistance. However, no group difference in reaction time task performance was found.

### Joint position sense–position control

The joint repositioning errors at 30° and 50° of knee flexion were more than twice as high in patients compared with healthy subjects. This finding is broadly consistent with various studies that have reported impaired proprioceptive accuracy for joint position sense in KOA patients [9]. The discharge of sensory receptors is modulated in and around the affected knee joint mainly due to swelling, pain, inflammation, joint laxity and damage to joint afferents. These modulations might induce changes in the central nervous system and thus activation of the quadriceps muscle (for a review see Rice et al. 2010) [53], resulting in decreased accuracy of torque generation and position sense [28].

Furthermore, an angle-specific difference in joint position sense between patients and control subjects was determined. Present results showed that patients produced greater error when the knee was in a more flexed position. These findings are in contrast to the results of Hortobagyi et al. [28] who found a greater repositioning error in more extended than flexed knee angles in KOA patients.

The reason for these contrary results may be due to the varying test protocols for measurement of joint position sense. Different protocols do not correlate well with each other and variations in protocol (e.g. sitting or lying position) seem to affect measurement outcome (for a review see Knoop 2011 [9]). Compared to many other studies, we measured joint position sense not in a sitting but in a lying position with a hip angle of 30°. In a lying position, the length of the biarticular rectus femoris muscle is increased compared to a sitting position accompanied by differences in moment arm and gravitational torques acting on the quadriceps muscle [12, 54]. Winter et al. have shown a high between-subject variability of the gradients of the rectus femoris force-length curves and speculated that this may require different activation strategies to produce optimally coordinated movements [12]. Furthermore, the results of van Ingen Schenau et al. suggest that motion and force-related feedback from peripheral afferents plays a more critical role in biarticular muscles [55]. It has been shown that different processes and sources of information are responsible for the control of mono- and biarticular muscles. Monoarticular muscles like the vastus medialis and vastus lateralis show simple flexor and extensor activation patterns, whereas biarticular muscles like the rectus femoris exhibit more flexible activation patterns and thus seem to play a greater role in the fine-regulation of joint moments of the limb [55].

### Reaction time task performance

Slow reaction times of the quadriceps muscle are associated with falls in older adults [14], but there is little research regarding this issue in KOA patients. No significant differences between patients and healthy controls in reaction time, premotor time and motor time were found, suggesting that the ability to react to simple visual cues and generate an appropriate motor response is not influenced by KOA. This finding is quite surprising as KOA is associated with higher fall risk [13] which in turn has been linked to decreased reaction time in elderly subjects [14].

### Isometric maximal voluntary contraction–isometric maximal voluntary force/torque and neuromuscular activation

Previous studies of patients with KOA have consistently observed quadriceps muscle strength deficits of up to 48% [16–19]. The results of this study indicate a torque-deficit during IMVC of 42% in patients with end-stage KOA. Quadriceps muscle weakness is attributed partly to muscle atrophy and partly to AMI [20]. Morphological changes of the quadriceps muscle include a selective atrophy of type II muscle fibres [56–58] resulting in a decrease in cross-sectional area of up to 12% compared to healthy subjects [59], which may be primarily a consequence of pain-related impaired physical mobility (disuse atrophy) [60]. There is further evidence of structural modulations, such as neurogenic muscle atrophy, and muscle fiber degeneration and regeneration [56]. Furthermore, changes in intra- and periarticular soft tissues alter the discharge of sensory receptors, which induces modulations in the central nervous system and the excitability of motor neurons [53, 61]. These modulations prevent the quadriceps muscle from being fully activated [20, 53, 61], resulting in a persistent quadriceps muscle weakness [16–19], which also promotes muscle atrophy [56, 59, 62, 63]. Consequently, quadriceps muscle weakness is directly and indirectly affected by AMI (for a review regarding AMI see Rice et al. 2010) [53]. The present data further indicate that lower IMVT was accompanied by a tendency toward lower activity of the quadriceps muscle. This difference, however, reached statistical significance only for the rectus femoris muscle. The data obtained are broadly consistent with other studies that have shown lower values for rectus femoris and vastus medialis muscle activity and no difference in vastus lateralis activity compared with healthy controls [24, 25]. The reduction in EMG activity and muscle atrophy might be an adaptation to long-term disuse of the affected leg due to KOA [64]. However, muscle activity of the quadriceps muscle was also found to be higher in two different knee flexion angles (30° and 60°) during IMVC in KOA patients [23]. The small number of studies and the contradictory findings indicate that further research on neuromuscular activation during IMVC is required.

### Isometric submaximal voluntary contraction–force/torque control and EMG-force/torque relationship

Our results show that the quality of torque production during isometric submaximal voluntary contractions was impaired in KOA patients.

Firstly, KOA patients exhibit a tendency toward lower torque accuracy (MAE) at 20% of IMVT whereas no differences were found at higher torque levels. These findings are partially in line with a previous study on force control in KOA patients. Hortobagyi et al. have shown that force accuracy is generally improved with increasing force level during dynamic contractions; however, no differences in MAE were found for isometric submaximal voluntary contractions [28]. The most likely explanation for the varying results compared to the present study is the application of different methods for the estimation of force/torque control. Hortobagyi et al. investigated force control during a force target-tracking task at two fixed force levels (50 N and 100 N). However, these determined target forces did not represent the same relative target forces in % of IMVC strength. The 50 N force level during isometric contractions corresponded to approximately 17% of IMVC strength in KOA patients and 10% of IMVC strength in control subjects. At a target force of 100 N, patients produced around 33% of IMVC strength and the control subjects 20%. Thus, these results should be interpreted with caution, as the comparability between groups remains difficult.

Secondly, it has been found that absolute torque fluctuation (SD) increased with higher target torques and differed between patients and control subjects at all three torque levels. In contrast, Hortobagyi et al. revealed unimpaired absolute force fluctuation during isometric submaximal voluntary contractions in KOA patients [28]. These varying results might be related to differences in the study protocol as discussed above. However, after absolute torque fluctuation data were normalized to torque and expressed as CV, no significant group-differences were documented, i.e. patients and control subjects did not differ in the amplitude of torque production. Only Smith et al. investigated relative force fluctuation in KOA patients; however, during dynamic but not isometric contractions. The authors found higher CV values at 50% of IMVC strength during concentric and eccentric voluntary contractions in patients compared to controls [35]. Nevertheless, present data indicate that in both groups the CV exhibits the greatest value at the low force level (20% IMVT) which is in line with findings in healthy subjects (for a review see Enoka 2003) [30]. Furthermore, there is a slightly–yet not significantly–higher CV in patients at 20% of IMVT. Differences in CV between young and elderly subjects have been reported at very low force levels, i.e. 2%, 5% and 10% of IMVT [29]. Thus, it can be speculated that relative force fluctuation might be higher in KOA patients compared to healthy controls at force levels lower than 20% of IMVT.

Thirdly, for the first time, the periodicity of the torque signal (MNF) was analyzed in KOA patients, and the results indicate that patients realized the same amplitude of torque control at 60% of IMVT with a higher frequency, i.e. torque tracking at a higher torque level required higher frequency responses.

Fourthly, the data indicate that more activity of the vastus lateralis is required to achieve low and moderate torque levels (20% and 40% of IMVT) whereas antagonist muscle activity was decreased at all torque intensities. Only Ling et al. and Berger et al. have evaluated muscle activity, motor unit recruitment and rate coding strategies of the quadriceps muscle at different submaximal force intensities [18, 26]. Ling et al. have shown that muscle activity of the vastus medialis was significantly lower in KOA patients with a higher Kellgren-Lawrence grade than in controls at 30% and 50% of IMVC whereas no significant differences were observed at lower intensities (10% and 20% of IMVC). The authors further demonstrated that patients with severe KOA recruited larger motor units (particularly at lower force levels) and a greater number of motor units to achieve different submaximal force levels compared to controls [18]. Nevertheless, these findings contradict each other because lower muscle activity is usually an indicator of reduced motor unit recruitment. In contrast, Berger et al. reported a recruitment of larger motor units of the vastus medialis and a reduced firing rate at 20% of IMVC compared to healthy age-matched controls [18, 26]. The varying results could be due to the use of different methodologies for identifying motor units. However, the recruitment of larger motor units at submaximal force levels possibly contributed to the greater muscle activity of the vastus lateralis at 20% and 40% of IMVT in the patient group in the present study. A second explanation for the altered EMG-torque relationship might be a differences in co-contraction of the hamstring muscles which was found to be higher in KOA patients [17]. A higher antagonist co-activation may require a greater activation of the agonist to produce the target force. Conversely, the present results indicate a lower biceps femoris activity. These results should, however, be viewed critically as an appropriate EMG normalization of the knee flexor muscle activity to that obtained during maximum knee flexion contraction was not possible. The measurement system can only be used for knee extension measurement. Thus, a meaningful evaluation of co-contraction was not possible; this is a limitation of the present study.

### Isometric submaximal fatiguing voluntary contraction–performance fatigability

The time to task failure during isometric submaximal fatiguing voluntary contraction at 40% of IMVT was twice as high in control subjects compared to KOA patients indicating a reduced fatigue resistance. To the best of our knowledge, only two studies have investigated performance fatigability of the quadriceps muscle in KOA patients [37, 38]. Fisher et al. studied fatigability during a sustained maximal voluntary contraction and reported a decrease in force of around 25% after a 90 s period [37]. Elboim-Gabyzon et al. analyzed the decrement in torque production following ten repeated IMVCs between KOA patients and controls [38]. The authors showed a reduction in IMVT of 14.4% in the involved leg and further demonstrated that fatigability in the involved leg was significantly lower than in the contralateral leg which was a quite unexpected finding. It was speculated that muscle fibre composition may differ between the legs. Higher pain und reduced mobility of the involved leg due to KOA may cause an asymmetric decline in type II fibres [56] resulting in a higher proportion of slow, fatigue-resistant type I fibres in the involved leg.

However, despite possible higher proportion of fatigue-resistant type I fibres, performance fatigability was increased in KOA patients. The cause of increased performance fatigability is multifactorial and may arise not only because of peripheral modulations of the involved muscles, but also because of changes in the central nervous system [65]. Research showed that voluntary activation of muscles can be impaired during submaximal fatiguing voluntary contractions [36, 66, 67]. It can be speculated that disease-related alterations of muscle afferent firing modulates motoneuronal output and thus might contribute to a higher voluntary activation deficit resulting in increased fatigability [67].

Summing up the results, it can be concluded that fatigue resistance is impaired during isometric submaximal voluntary contractions of the quadriceps muscle in KOA. However, further research is required to clarify the neuromuscular mechanisms behind lower performance fatigability.

### Limitations

Post-hoc power analysis for non-significant and tendentially significant results revealed low power coefficients (power < 0.80) indicating an increased risk of a type II error. The lack of power, possibly related to small sample size, might be an alternative explanation for non-significant findings. Tendentially significant differences could have been significant when the study had been adequately powered.

## Conclusion

The present study evaluated the underlying neuromuscular mechanisms of quadriceps muscle dysfunction and provided important information for optimizing rehabilitation exercises for the treatment of KOA. Neuromuscular function of the quadriceps muscle was impaired during isometric maximal, submaximal and submaximal fatiguing voluntary contractions in patients with end-stage KOA. The present data have shown that KOA is not only associated with a deterioration of torque and neuromuscular activation during IMVC, but also with an impaired position and torque control at submaximal torque levels, an altered EMG-torque relationship and a higher fatigability of the quadriceps muscle. However, simple reaction time task performance was not significantly different between KOA patients and healthy controls. On the basis of the results of this study, it is recommended that the rehabilitation include strengthening and fatiguing exercises at maximal and submaximal force levels during all stages of KOA in order to improve function, reduce mobility limitations and slow progression of disease.

## Supporting information

S1 Dataset Raw data. (XLSX)

## References

[1] Hurley MV. The role of muscle weakness in the pathogenesis of osteoarthritis. Rheum Dis Clin North Am. 1999;25(2):283–98, vi. 10356418 10.1016/s0889-857x(05)70068-5

[2] Bennell KL, Wrigley TV, Hunt MA, Lim BW, Hinman RS. Update on the role of muscle in the genesis and management of knee osteoarthritis. Rheum Dis Clin North Am. 2013;39(1):145–76. doi: 10.1016/j.rdc.2012.11.003 23312414 10.1016/j.rdc.2012.11.003

[3] Zacharias A, Green RA, Semciw AI, Kingsley MI, Pizzari T. Efficacy of rehabilitation programs for improving muscle strength in people with hip or knee osteoarthritis: a systematic review with meta-analysis. Osteoarthritis Cartilage. 2014;22(11):1752–73. doi: 10.1016/j.joca.2014.07.005 25065642 10.1016/j.joca.2014.07.005

[4] Fransen M, McConnell S, Harmer AR, Van der Esch M, Simic M, Bennell KL. Exercise for osteoarthritis of the knee. Cochrane Database Syst Rev. 2016;1:CD004376. 10.1002/14651858.CD004376.pub3 PMC10094004 25569281

[5] Tanaka R, Ozawa J, Kito N, Moriyama H. Effects of exercise therapy on walking ability in individuals with knee osteoarthritis: a systematic review and meta-analysis of randomised controlled trials. Clin Rehabil. 2015;30(1):36–52. doi: 10.1177/0269215515570098 25691583 10.1177/0269215515570098

[6] Mizner RL, Petterson SC, Stevens JE, Axe MJ, Snyder-Mackler L. Preoperative quadriceps strength predicts functional ability one year after total knee arthroplasty. J Rheumatol. 2005;32(8):1533–9. 16078331

[7] Nelson AE, Allen KD, Golightly YM, Goode AP, Jordan JM. A systematic review of recommendations and guidelines for the management of osteoarthritis: The chronic osteoarthritis management initiative of the U.S. bone and joint initiative. Semin Arthritis Rheum. 2014;43(6):701–12. doi: 10.1016/j.semarthrit.2013.11.012 24387819 10.1016/j.semarthrit.2013.11.012

[8] Takacs J, Carpenter MG, Garland SJ, Hunt MA. The role of neuromuscular changes in aging and knee osteoarthritis on dynamic postural control. Aging Dis. 2013;4(2):84–99. 23696951 PMC3659254

[9] Knoop J, Steultjens MP, van der Leeden M, van der Esch M, Thorstensson CA, Roorda LD, et al Proprioception in knee osteoarthritis: a narrative review. Osteoarthritis Cartilage. 2011;19(4):381–8. doi: 10.1016/j.joca.2011.01.003 21251988 10.1016/j.joca.2011.01.003

[10] Lewek MD, Schmit BD, Hornby G, Dhaher YY. Hip joint position modulates volitional knee extensor muscle activity after stroke. Muscle Nerve. 2006;34:767–74. doi: 10.1002/mus.20663 16967491 10.1002/mus.20663

[11] Watanbe K, Kouzaki M, Moritani T. Non uni-form surface electromyographic response to change in joint angle within rectus femoris muscle. Muscle Nerve. 2014;50(794–802). doi: 10.1002/mus.24232 24590732 10.1002/mus.24232

[12] Winter SL, Challis JH. The force-length curves of the human rectus femoris and gastrocnemius muscles in vivo. Journal of Applied Biomechanics. 2010;26:45–51. 20147757 10.1123/jab.26.1.45

[13] Levinger P, Menz HB, Wee E, Feller JA, Bartlett JR, Bergman NR. Physiological risk factors for falls in people with knee osteoarthritis before and early after knee replacement surgery. Knee Surg Sports Traumatol Arthrosc. 2011;19(7):1082–9. doi: 10.1007/s00167-010-1325-8 21107530 10.1007/s00167-010-1325-8

[14] Lajoie Y, Gallagher SP. Predicting falls within the elderly community: comparison of postural sway, reaction time, the Berg balance scale and the Activities-specific Balance Confidence (ABC) scale for comparing fallers and non-fallers. Arch Gerontol Geriatr. 2004;38(1):11–26. 14599700 10.1016/s0167-4943(03)00082-7

[15] Roos EM, Herzog W, Block JA, Bennell KL. Muscle weakness, afferent sensory dysfunction and exercise in knee osteoarthritis. Nat Rev Rheumatol. 2011;7(1):57–63. doi: 10.1038/nrrheum.2010.195 21119605 10.1038/nrrheum.2010.195

[16] Gapeyeva H, Buht N, Peterson K, Ereline J, Haviko T, Paasuke M. Quadriceps femoris muscle voluntary isometric force production and relaxation characteristics before and 6 months after unilateral total knee arthroplasty in women. Knee Surg Sports Traumatol Arthrosc. 2007;15(2):202–11. doi: 10.1007/s00167-006-0166-y 17006663 10.1007/s00167-006-0166-y

[17] Stevens-Lapsley JE, Balter JE, Kohrt WM, Eckhoff DG. Quadriceps and hamstrings muscle dysfunction after total knee arthroplasty. Clin Orthop Relat Res. 2010;468(9):2460–8. doi: 10.1007/s11999-009-1219-6 20087703 10.1007/s11999-009-1219-6 PMC2919870

[18] Ling SM, Conwit RA, Talbot L, Shermack M, Wood JE, Dredge EM, et al Electromyographic patterns suggest changes in motor unit physiology associated with early osteoarthritis of the knee. Osteoarthritis Cartilage. 2007;15(10):1134–40. doi: 10.1016/j.joca.2007.03.024 17543548 10.1016/j.joca.2007.03.024 PMC2259251

[19] Bade MJ, Kohrt WM, Stevens-Lapsley JE. Outcomes before and after total knee arthroplasty compared to healthy adults. J Orthop Sports Phys Ther. 2010;40(9):559–67. doi: 10.2519/jospt.2010.3317 20710093 10.2519/jospt.2010.3317 PMC3164265

[20] Pietrosimone BG, Hertel J, Ingersoll CD, Hart JM, Saliba SA. Voluntary Quadriceps Activation Deficits in Patients with Tibiofemoral Osteoarthritis: A Meta-Analysis. Physical medicine and rehabilitation. 2011;3:153–62. 10.1016/j.pmrj.2010.07.485 21333954

[21] Mizner RL, Petterson SC, Snyder-Mackler L. Quadriceps strength and the time course of functional recovery after total knee arthroplasty. J Orthop Sports Phys Ther. 2005;35(7):424–36. doi: 10.2519/jospt.2005.35.7.424 16108583 10.2519/jospt.2005.35.7.424

[22] Ploutz-Snyder LL, Manini T, Ploutz-Snyder RJ, Wolf DA. Functionally relevant thresholds of quadriceps femoris strength. J Gerontol A Biol Sci Med Sci. 2002;57(4):B144–52. 11909879 10.1093/gerona/57.4.b144

[23] Marks R, Percy JS, Semple J, Kumar S. Comparison between the surface electromyogram of the quadriceps surrounding the knees of healthy women and the knees of women with osteoarthrosis. Clin Exp Rheumatol. 1994;12(1):11–5. 8162635

[24] Hiranaka T, Takeuchi K. Electromyogaphic findings in muscles around the osteoarthritic knee: integrated electromyography and frequency analysis. Nihon Seikeigeka Gakkai Zasshi. 1995;69(9):675–84. 8530883

[25] Rice DA, McNair PJ, Lewis GN. Mechanisms of quadriceps muscle weakness in knee joint osteoarthritis: the effects of prolonged vibration on torque and muscle activation in osteoarthritic and healthy control subjects. Arthritis Res Ther. 2011;13(5):R151 doi: 10.1186/ar3467 21933392 10.1186/ar3467 PMC3308081

[26] Berger MJ, Chess DG, Doherty TJ. Vastus medialis motor unit properties in knee osteoarthritis. BMC Musculoskelet Disord. 2011;12:199 doi: 10.1186/1471-2474-12-199 21910917 10.1186/1471-2474-12-199 PMC3182963

[27] Hortobagyi T, Tunnel D, Moody J, Beam S, DeVita P. Low- or high-intensity strength training partially restores impaired quadriceps force accuracy and steadiness in aged adults. J Gerontol A Biol Sci Med Sci. 2001;56(1):B38–47. 11193224 10.1093/gerona/56.1.b38

[28] Hortobagyi T, Garry J, Holbert D, Devita P. Aberrations in the control of quadriceps muscle force in patients with knee osteoarthritis. Arthritis Rheum. 2004;51(4):562–9. doi: 10.1002/art.20545 15334428 10.1002/art.20545

[29] Tracy BL, Enoka RM. Older adults are less steady during submaximal isometric contractions with the knee extensor muscles. J Appl Physiol (1985). 2002;92(3):1004–12. 11842033 10.1152/japplphysiol.00954.2001

[30] Enoka RM, Christou EA, Hunter SK, Kornatz KW, Semmler JG, Taylor AM, et al Mechanisms that contribute to differences in motor performance between young and old adults. J Electromyogr Kinesiol. 2003;13(1):1–12. 12488083 10.1016/s1050-6411(02)00084-6

[31] Singh NB, Arampatzis A, Duda G, Heller MO, Taylor WR. Effect of fatigue on force fluctuations in knee extensors in young adults. Philos Trans A Math Phys Eng Sci. 2010;368(1920):2783–98. doi: 10.1098/rsta.2010.0091 20439273 10.1098/rsta.2010.0091

[32] Singh NB, Konig N, Arampatzis A, Heller MO, Taylor WR. Extreme levels of noise constitute a key neuromuscular deficit in the elderly. PLoS One. 2012;7(11):e48449 doi: 10.1371/journal.pone.0048449 23139783 10.1371/journal.pone.0048449 PMC3491054

[33] Singh NB, Konig N, Arampatzis A, Taylor WR. Age-related modifications to the magnitude and periodicity of neuromuscular noise. PLoS One. 2013;8(12):e82791 doi: 10.1371/journal.pone.0082791 24349362 10.1371/journal.pone.0082791 PMC3861468

[34] Seynnes O, Hue OA, Garrandes F, Colson SS, Bernard PL, Legros P, et al Force steadiness in the lower extremities as an independent predictor of functional performance in older women. J Aging Phys Act. 2005;13(4):395–408. 16301752 10.1123/japa.13.4.395

[35] Smith JW, Marcus RL, Peters CL, Pelt CE, Tracy BL, LaStayo PC. Muscle force steadiness in older adults before and after total knee arthroplasty. J Arthroplasty. 2014;29(6):1143–8. doi: 10.1016/j.arth.2013.11.023 24405624 10.1016/j.arth.2013.11.023

[36] Gandevia SC. Spinal and supraspinal factors in human muscle fatigue. Physiol Rev. 2001;81(4):1725–89. 11581501 10.1152/physrev.2001.81.4.1725

[37] Fisher NM, White SC, Yack HJ, Smolinski RJ, Pendergast DR. Muscle function and gait in patients with knee osteoarthritis before and after muscle rehabilitation. Disabil Rehabil. 1997;19(2):47–55. 9058029 10.3109/09638289709166827

[38] Elboim-Gabyzon M, Rozen N, Laufer Y. Quadriceps femoris muscle fatigue in patients with knee osteoarthritis. Clin Interv Aging. 2013;8:1071–7. doi: 10.2147/CIA.S42094 23976847 10.2147/CIA.S42094 PMC3746781

[39] Bijur PE, Silver W, Gallagher EJ. Reliability of the visual analog scale for measurement of acute pain. Acad Emerg Med. 2001;8(12):1153–7. 11733293 10.1111/j.1553-2712.2001.tb01132.x

[40] Jerosch J, Prymka M. [Proprioceptive capacities of the healthy knee joint: modification by an elastic bandage]. Sportverletz Sportschaden. 1995;9(3):72–6. doi: 10.1055/s-2007-993428 7502216 10.1055/s-2007-993428

[41] Barrett DS, Cobb AG, Bentley G. Joint proprioception in normal, osteoarthritic and replaced knees. J Bone Joint Surg Br. 1991;73(1):53–6. 1991775 10.1302/0301-620X.73B1.1991775

[42] Lin DH, Lin CH, Lin YF, Jan MH. Efficacy of 2 non-weight-bearing interventions, proprioception training versus strength training, for patients with knee osteoarthritis: a randomized clinical trial. J Orthop Sports Phys Ther. 2009;39(6):450–7. doi: 10.2519/jospt.2009.2923 19531879 10.2519/jospt.2009.2923

[43] Botwinick J, Thompson LW. Premotor and motor components of reaction time. J Exp Psychol. 1966;71(1):9–15. 5902149 10.1037/h0022634

[44] Behrens M, Mau-Moeller A, Wassermann F, Bader R, Bruhn S. Effect of balance training on neuromuscular function at rest and during isometric maximum voluntary contraction. Eur J Appl Physiol. 2015;115(5):1075–85. doi: 10.1007/s00421-014-3089-1 25557387 10.1007/s00421-014-3089-1

[45] Behrens M, Mau-Moeller A, Weippert M, Fuhrmann J, Wegner K, Skripitz R, et al Caffeine-induced increase in voluntary activation and strength of the quadriceps muscle during isometric, concentric and eccentric contractions. Sci Rep. 2015;5:10209 doi: 10.1038/srep10209 25969895 10.1038/srep10209 PMC4429543

[46] Behrens M, Mau-Moeller A, Mueller K, Heise S, Gube M, Beuster N, et al Plyometric training improves voluntary activation and strength during isometric, concentric and eccentric contractions. J Sci Med Sport. 2016;19(2):170–6. doi: 10.1016/j.jsams.2015.01.011 25766509 10.1016/j.jsams.2015.01.011

[47] Alkner BA, Tesch PA, Berg HE. Quadriceps EMG/force relationship in knee extension and leg press. Med Sci Sports Exerc. 2000;32(2):459–63. 10694132 10.1097/00005768-200002000-00030

[48] Cohen J. Statistical Power Analysis for the Behavioral Sciences. Hillsdale, New Jersey: Lawrence Erlbaum Assoc Inc; 1988.

[49] Atkinson G, Nevill AM. Statistical methods for assessing measurement error (reliability) in variables relevant to sports medicine. Sports Med. 1998;26(4):217–38. 9820922 10.2165/00007256-199826040-00002

[50] Stokes M. Reliability and repeatability of methods for measuring muscle in physiotherapy. Physiother Pract. 1985;1:71–6.

[51] Vincent WJ. Statistics in Kinesiology. Champaign, IL: Human Kinetics; 1999. 294 p.

[52] Hopkins WG. Precision of measurement. In: A New View of Statistics (newstats.org/precision.html)2011.

[53] Rice DA, McNair PJ. Quadriceps arthrogenic muscle inhibition: neural mechanisms and treatment perspectives. Semin Arthritis Rheum. 2010;40(3):250–66. doi: 10.1016/j.semarthrit.2009.10.001 19954822 10.1016/j.semarthrit.2009.10.001

[54] Worringham CJ, Stelmach GE. The contribution of gravitational torques to limb position sense. Exp Brain Res. 1985;61(1):38–42. 4085602 10.1007/BF00235618

[55] van Ingen Schenau GJ, Pratt CA, Macpherson JM. Differential use and control of mono- and biarticular muscles. Human Movement Science. 1994;13:495–517.

[56] Fink B, Egl M, Singer J, Fuerst M, Bubenheim M, Neuen-Jacob E. Morphologic changes in the vastus medialis muscle in patients with osteoarthritis of the knee. Arthritis Rheum. 2007;56(11):3626–33. doi: 10.1002/art.22960 17968889 10.1002/art.22960

[57] Nakamura T, Suzuki K. Muscular changes in osteoarthritis of the hip and knee. Nihon Seikeigeka Gakkai Zasshi. 1992;66(5):467–75. 1387155

[58] Glasberg MR, Glasberg JR, Jones RE. Muscle pathology in total knee replacement for severe osteoarthritis: a histochemical and morphometric study. Henry Ford Hosp Med J. 1986;34(1):37–40. 3700127

[59] Ikeda S, Tsumura H, Torisu T. Age-related quadriseps-dominant muscle atrophy and incident radiographic knee osteoarthritis. I Orthop Sci. 2005;10:121–6. 10.1007/s00776-004-0876-2 15815857

[60] Tanaka S, Hachisuka K, Nara S, Ogata H, Kobayashi Y, Tanaka H. Effect of activities of daily living on fiber type atrophy of the vastus lateralis muscle in patients with joint disorders. Am J Phys Med Rehabil. 1998;77(2):122–7. 9558013

[61] Hurley MV, Scott DL, Rees J, Newham DJ. Sensorimotor changes and functional performance in patients with knee osteoarthritis. Ann Rheum Dis. 1997;56(11):641–8. 9462165 10.1136/ard.56.11.641 PMC1752287

[62] Petterson SC, Barrance P, Buchanan T, Binder-Macleod S, Snyder-Mackler L. Mechanisms underlying quadriceps weakness in knee osteoarthritis. Med Sci Sports Exerc. 2008;40(3):422–7. doi: 10.1249/MSS.0b013e31815ef285 18379202 10.1249/MSS.0b013e31815ef285 PMC3573845

[63] Mizner RL, Petterson SC, Stevens JE, Vandenborne K, Snyder-Mackler L. Early quadriceps strength loss after total knee arthroplasty. The contributions of muscle atrophy and failure of voluntary muscle activation. J Bone Joint Surg Am. 2005;87(5):1047–53. doi: 10.2106/JBJS.D.01992 15866968 10.2106/JBJS.D.01992 PMC1167681

[64] Suetta C, Aagaard P, Magnusson SP, Andersen LL, Sipila S, Rosted A, et al Muscle size, neuromuscular activation, and rapid force characteristics in elderly men and women: effects of unilateral long-term disuse due to hip-osteoarthritis. J Appl Physiol (1985). 2007;102(3):942–8. 17122381 10.1152/japplphysiol.00067.2006

[65] Enoka RM, Duchateau J. Translating fatigue to human performance. Medicine and Science in Sports and Exercise. 2016;48(11):2228–38. doi: 10.1249/MSS.0000000000000929 27015386 10.1249/MSS.0000000000000929 PMC5035715

[66] Enoka RM, Baudry S, Rudroff T, Farina D, Klass M, Duchateau J. Unraveling the neurophysiology of muscle fatigue. J Electromyogr Kinesiol. 2011;21(2):208–19. doi: 10.1016/j.jelekin.2010.10.006 21071242 10.1016/j.jelekin.2010.10.006

[67] Taylor JL, Gandevia SC. A comparison of central aspects of fatigue in submaximal and maximal voluntary contractions. J Appl Physiol. 2008;104(2):542–50. doi: 10.1152/japplphysiol.01053.2007 18032577 10.1152/japplphysiol.01053.2007

